# Preparation of Losartan Potassium Controlled Release Matrices and In-Vitro Investigation Using Rate Controlling Agents

**DOI:** 10.3390/molecules27030864

**Published:** 2022-01-27

**Authors:** Kamran Ahmad Khan, Gul Majid Khan, Muhammad Muzammal, Mohammed Al Mohaini, Abdulkhaliq J. Alsalman, Maitham A. Al Hawaj, Ashfaq Ahmad, Zahid Rasul Niazi, Kifayat Ullah Shah, Arshad Farid

**Affiliations:** 1 Gomal Centre of Pharmaceutical Sciences, Faculty of Pharmacy Gomal University, Dera Ismail Khan 29050, Pakistan; dr.kamran.gu@gmail.com (K.A.K.); zahidscholar1@gmail.com (Z.R.N.); Kifayatrphr@gmail.com (K.U.S.); 2 Department of Pharmacy, Quaid-i-Azam University, Islamabad 45320, Pakistan; drgulmajeed@yahoo.com; 3 Gomal Center of Biochemistry and Biotechnology, Gomal University, Dera Ismail Khan 29050, Pakistan; mustafamuzammal1@yahoo.com; 4 Basic Sciences Department, College of Applied Medical Sciences, King Saud Bin Abdulaziz University for Health Sciences, Alahsa 31982, Saudi Arabia; mohainim@ksau-hs.edu.sa; 5 King Abdullah International Medical Research Center, Alahsa 31982, Saudi Arabia; 6 Department of Clinical Pharmacy, Faculty of Pharmacy, Northern Border University, Rafha 91911, Saudi Arabia; KALIQS@gmail.com; 7 Department of Pharmacy Practice, College of Clinical Pharmacy, King Faisal University, Alahsa 31982, Saudi Arabia; hawaj@kfu.edu.sa; 8 Department of Pharmacy, University of Swabi, Swabi 23430, Pakistan; ashfaqahmad@uoswabi.edu.pk

**Keywords:** direction compression method, losartan potassium, development, release kinetic, dissolution profile

## Abstract

Controlled release matrices have predictable drug release kinetics, provide drugs for an extended period of time, and reduce dosing frequency with improved patient compliance as compared with conventional tablet dosage forms. In the current research work, losartan potassium controlled release matrix tablets were fabricated and prepared with rate altering agents; that is, Ethocel grade 100 combined with Carbopol 934PNF. Various drug to polymer ratios were used. HPMC, CMC, and starch were incorporated in some of the matrices by replacing some amount of filler (5%). The direct compression method was adopted for the preparation of matrices. In phosphate buffer (pH 6.8), the dissolution study was conducted by adopting the USP method-I as the specified method. Drug release kinetics was determined and dissolution profiles were also compared with the reference standard. Prolonged release was observed for all matrices, but those with Ethocel 100FP Premium showed more extended release. The co-excipient (HPMC, CMC, and starch) exhibited enhancement in the drug release rates, while all controlled release matrices released the drug by anamolous non-Fickian diffusion mechanism. This combination of polymers (Ethocel grade 100 with Carbopol 934PNF) efficiently extended the drug release rates up to 24 h. It is suggested that these matrix tablets can be given in once a day dosage, which might improve patient compliance, and the polymeric blend of Ethocel grade 100 with Carbopol 934PNF might be used in the development of prolonged release matrices of other water-soluble drugs.

## 1. Introduction

The delivery of drugs has changed over time with drugs targeting specific tissues like cancer tissue or sustained and controlled rates of drug delivery [1]. Nowadays, novel drug delivery systems are continuously replacing conventional drug delivery systems. Recently, controlled release systems have been tremendously popular. They avoid multiple dosing and as well as prolonged delivery of drugs, which has importance for scientists as well the pharmaceutical industry [2]. Controlled release (CR) systems offer constant release for a longer duration with improved compliance [3]. A perfect drug delivery system has two good basic aspects; that is, providing the required drug content and being target-specific. Conventional and controlled release dosage forms have the same systemic availability as well as therapeutic effects when prepared in different dosages, but the only difference observed was the single dosage for controlled release forms [4]. It is well known that controlled release devices have predictability and reproducibility in release kinetics [5]. In another study, metformin HCl matrices were developed with various polymers to sustain the drug release rates [6]. Flurbiprofen controlled release matrix tablets were prepared to extend the drug release rates, with Eudragit as a rate-controlling agent [7]. Matrix tablets are well-known controlled release dosage forms, releasing the drug either by dissolution or diffusion mechanism. Drug and rate-controlling agents are mixed homogeneously, and rate-controlling agents can be hydrophilic, mineral, lipid, or plastic, among others [8]. Carbamazepine controlled release tablets were also developed with polymers such as HPMC of various grades, using the wet granulation technique and some using the direct compression method. They found that the drug was efficiently extended by HPMC [9]. Glipizide controlled release matrices were prepared by direct compression technique and used Eudragit and HPMC as polymers, and evaluated its physicochemical characteristics and noted that drug release was extended [10]. Losartan potassium belongs to the group of angiotensin 2 receptor blockers and is mostly used in the management of high blood pressure. Its half-life is about 2 h and it is available in off-white crystalline powder [11]. It is freely soluble in phosphate buffer 6.8 pH [12]. Sustained release losartan potassium matrices were developed by the direct compression method using polymers ethylcellulose, eudragit RSPO, and eudragit RLPO, and it was noted that drug release rates were more extended with ethylcellulose when used in combination than polymers used alone [13]. The authors of [14] prepared sustained release matrix tablets of losartan potassium with xanthan gum by direct compression methods and evaluated the in vitro dissolution as well pharmacokinetics. In another study, controlled release matrices were developed with synthetic and non-synthetic polymers and evaluated for physic-chemical characteristic, and it was found that polymeric combination attained 24 h release of the drug [15]. The authors of [16] developed sustained release matrices of losartan potassium with gum prosophis juliflora as a rate-altering agent, and the authors noted that the polymeric material sustained the drug release rates. Losartan potassium sustained release matrices were prepared with xanthan gum, ethylcellulose, and HPMC and evaluated for in vitro dissolution, and it was observed that formulation F3 sustained drug release rates up to 10 h [17]. Directly compressed controlled release matrices of losartan potassium were prepared with the following polymers: sodium alginate, pectin, and xanthan gum, and dissolution studies were performed for drug release. It was noted that drug release was in controlled fashion from the matrices [18]. Losartan potassium controlled release matrices were prepared using ethocel grade 7 and carbopol combination and were evaluated for in vitro release study; it was noted that this polymeric combination controlled the drug release rates [19]. In the current study, losartan potassium controlled release matrices were developed with ethocel grade 100 and carbopol 934P NF, and it was noted that this new polymer combination more efficiently extended drug release rates up to 24 h, and that it might be given in a once a day dosage with improved patient compliance.

## 2. Materials and Methods

### 2.1. Materials

Dissolution apparatus (PTWS-11/P, Hunburg, Germany), Carbopol 934P NF (Lubrizol, Wickliffe, OH, USA), and losartan potassium were donated by Well & Well Pharmaceutical of Pakistan, Ethocel grade 100 (Dow Chemical Co., Midland, MI, USA). Chemicals were of analytical quality and used without any further purification.

### 2.2. Tablets’ Fabrication

Ethocel grade 100 was combined with carbopol 934P NF using various ratios of 10:3, 10:4, and 10:5. The drug among was constant in all the formulated tablets, but the amount of polymers varied, and filler and lubricant were also part of the formulations as given in Table 1. One-hundred tablets were fabricated and formulated as the pilot batch.

### 2.3. Flow Properties

In the development of a good tablet product, the determination of flow properties is a very important aspect. It can be measured from parameters of flow such as the angle of repose (θ = tan^− 1^ h/r), Carr’s index [(Vo − Vf/Vo) × 100] [20], and Hausner’s ratio. The parameters were determined for each formulation mixture according to standard procedures [20,21].

### 2.4. Tablets’ Preparation

All ingredients were weighed and the polymer and drug were mixed using pestle and mortar. To this mixture, other excipients were added mixed and geometrically and passed through screen no. 32. This mixture was lubricated with magnesium stearate and passed twice again through same screen to remove any particulate material, and compression was done with a tableting machine with a hardness of 5–10 kg/cm^2^.

### 2.5. Physical Characteristic

Various tests like diameter, hardness, thickness, friability, and weight variation were performed according to standard procedures adopted by other researchers. Thickness and diameters were measured in mm, while friability was measured in percent and harness in kg/cm^2^, as well as weight variation in milligrams. Tablets (*n* = 10) were taken and their thickness and diameters were determined with vernier calliper (Erweka, Langen, Germany). Hardness of tablets (*n* = 10) was determined with a hardness tester. The friability of 20 tablets was determined with the use of a friabilator. In the weight variation test, tablets (*n* = 20) were taken and checked by weighing each individual tablet, and mean weights were determined with digital electronic balance (Japan) [20,22].

### 2.6. Dissolution

In dissolution apparatus, 900 mL of phosphate buffer at 6.8 pH was added, and the test was conducted by means of the USP method-I. Baskets were set at 100 rpm and temperature was maintained thermostatically at 37 ± 0.5 °C. Samples were drawn at desired time gaps and filtered with a membrane filter of 0.45 µm. Spectrophotometrically, the analysis of each sample was carried out and the absorbances (at λmax of 205 nm) were obtained. The drug and cumulative release was determined from a standard curve, while all experiments were performed in triplicate.

### 2.7. Drug Release Mechanisms

Drug release mechanisms were obtained by placing values of cumulative drug release rates into various mathematical models for drug release kinetics, such as zero-order, first-order, Hixon crowell’s [23], Highuchi [24], and power law models [25]. 

### 2.8. Difference and Similarity Factors

Dissolution comparison was done by applying difference and similarity factors, i.e., *f*_1_ and *f*_2_, respectively [26]. Cardaktin^®^ tablets were taken as a reference for comparison.

## 3. Results and Discussion

### 3.1. Flow Properties

Flow properties were noted for each batch of tablet formulation powders. The results were found to be within acceptable limits [20], and the results are mentioned in Table 2. As flow properties are required to know the flowability of the formulation mixtures, which can affect the preparation of tablets and good flow properties, one should avoid sticking with punches or die cavity; these findings are similar to those of authors [19], that physical characteristics influence the tablets’ design and development.

### 3.2. Physical Characteristics

Tablets were good in their appearance and the entire physical characteristics ranged within the acceptable limits [19], and the results are presented as mean ± SD in Table 3. These physical characteristics affect dissolution and release kinetics and, when these characteristics are in acceptable limits, they might give a good release profile and release kinetics. These findings are similar to those of other authors [19], that good physical characteristics result in quality tablets.

### 3.3. Drug Release from Tablets

**Figure 1:**
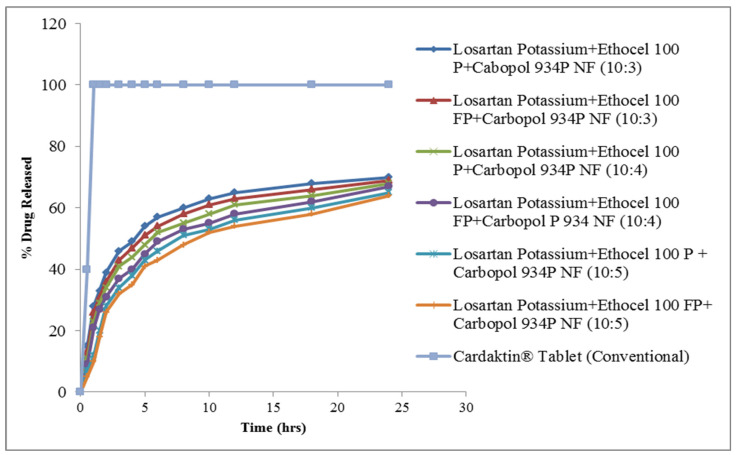
Release patterns of CR matrices of ethocel grade 100 and carbopol P 934 NF.

This polymeric blend in various amounts extended the drug release up to 24 h. Moreover, it was noted that ethocel grade 100 formulations further extended the drug release rates as compared with ethocel grade 7 with carbopol [19]. Premium formulation released 70, 68 and 65% of the drug and FP premium released 69, 67, and 64% of the drug, showing a little bit more retardation than premium polymer blended with carbopol. The results are given in Figure 1.

**Figure 2:**
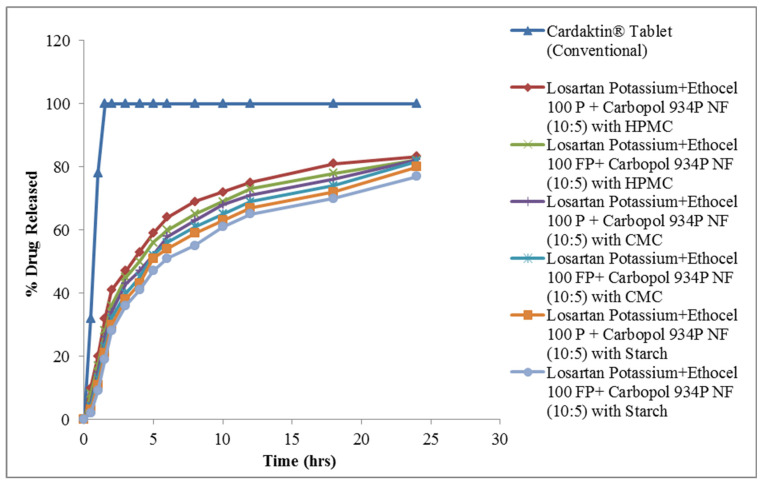
Drug release from polymeric matrices with co-excipients.

When co-excipient HPMC was added to the polymeric blend of Ethocel 100 Premium and Carbopol 934 Premium grade, the drug released occurring at 10:3 was 83.26% and 82.18%, respectively. For the same polymeric blend at 10:4, when CMC was added, the drug release rate was 83.26 and 81.77%, respectively. In this case, when starch was added at 10:5, an increase was noted and the drug release enhanced to 80% and 77%, respectively. The results are shown in Figure 2. The polymers controlled the drug release rates for 24 h. Ethocel 100 grade well prolonged the drug release rates when combined with carbopol. FP containing matrix tablets additionally prolonged the drug release rates as compared with 100 premium formulations, as FP was in fine particulate form. Ethocel 100 grade, being a hydrophobic polymer, retarded the water penetration and extended the drug released rates. As Ethocel 100 FP premium is available in fine particle, this caused further drug retardation as compared with simple ethocel 100 premiums, which exists in granular form. The current study findings are similar to those of other authors [19,21,27], that ethocel grade 100 well prolonged the release rate. When ethocel was used in combination with Carbopol, it led to retardation of the release of the drug that could be due to the hydrophobic nature of ethocel, and hydration of Carbopol causes a decrease in the micropores’ size and results in more retardation. The present findings are similar to the conclusions of other authors [19,28], that the combination of Ethocel with Carbopol retarded the drug release rates. A co-excipient like HPMC, when used in a small quantity and because it is water soluble, could increase the osmotic pressure in the matrices, which might result in an increase in the drug release rate from polymeric matrices. Similarly, CMC as well as starch, when used in small amounts acting as a disintegrant, results in increased drug release rates. The findings are similar to those of other authors [19,29], that HPMC, CMC, and starch act as disintegrates in small amounts and increase the drug release rates. These combinations were found to give better results in terms of drug retardation, and co-excipients increase release patterns. 

### 3.4. Content Uniformity

This test was carried out according to standard protocol, and the results of content uniformity [26] were found to be within USP specified limits as shown in Table 4.

### 3.5. Kinetics of Drug Release and Dissolution Comparison

When applying various kinetic models to accumulate drug release data, good results were noted. The results (mean ± SD) are shown in Table 5. The data well fitted the power law [16] and values of *n* were noted (0.626 to 0.957). This indicates that the drug was released by anamolous non-Fickian diffusion. Formulation with ethocel 100 premium and carbopol P 934NF showed a better result in the case of drug release that the *n* value (0.957) approached the ideal zero-order kinetics and the drug might be released by diffusion and erosion owing to hydrophilic and hydrophobic polymer combination. It was noted previously that drug release occurred by diffusion and erosion from polymeric tablets with carbopol [30]. Dissolution profiles of reference tablets (Cardaktin^®^ tablets) and test matrices were compared; the resultant f1 values ranged from 38.16 to 56.48 and f2 values ranged from 10.16 to 17.99. The results are given in Table 6. The drug release occurred by anamolous non-Fickian diffusion when applying the power law kinetic model [26] to drug release data, and there was no match observed between the dissolution profiles of tested and reference formulation when applying difference and similarity factors [27]. These polymers when used in combination might extend drug release rates in similar products. 

## 4. Conclusions

In current study, the effect of Ethocel grade 100 combined with Carbopol 934P NF was investigated, and excellent results were observed in terms of release retardation, extending the drug release rate up to 24 h. The drug release mechanism was anamolous non-Fickian and erosion. The drug release profiles of tested and reference matrices did not match with each other when applying difference and similarity factors. The co-excipients, HPMC, CMC, and starch, enhanced the drug release rate from these polymeric matrices. It can be concluded that these polymeric combinations might improve patient compliance and can be successfully used in the preparation of controlled release formulations of other drugs.

## Figures and Tables

**Table 1:** Composition of tablets.

Losartan Potassium CR Tablets					
D:P	Drug (mg)	Polymers’ Combination (Ethocel 100 Premium + Carbopol 934P NF and Ethocel 100 FP Premium + Carbopol 934P NF) (mg)		Filler (mg)	Magnesium Stearate 0.5% (mg)
10:3	100	30		69	1.0
10:4	100	40		59	1.0
10:5	100	50		49	1.0
Losartan Potassium CR tablets with Co-excipients					
D:P	Drug (mg)	Polymeric combination (mg)	Filler (mg)	Lubricant (0.5%)	Co-excipient (10% of filler of HPMC or CMC or Starch)
10:5	100	50	44.1	1.0	4.9 mg
Drug: Losartan potassium Filler: Spray dried lactose					

**Table 2:** Flow properties of formulation mixtures.

Formulations	Angle of Repose (*n* = 3, mean ± SD)	Carr’s Index (*n* = 3, mean ± SD)	Hausner’s Ratio (*n* = 3, mean ± SD)
Ethocel 100P + Carbopol 934P NF (10:3)	30.24 ± 0.65	11.64 ± 0.49	1.16 ± 0.51
Ethocel 100FP + Carbopol 934P NF (10:3)	33.61 ± 0.53	14.04 ± 0.44	1.17 ± 0.82
Ethocel 100P + Carbopol 934P NF (10:4)	29.59 ± 0.62	9.12 ± 0.28	1.10 ± 0.69
Ethocel 100FP + Carbopol 934P NF (10:4)	25.41 ± 0.30	9.1 ± 0.23	1.02 ± 0.81
Ethocel 100P + Carbopol 934P NF (10:5)	26.16 ± 0.06	10.04 ± 0.09	1.0 ± 0.11
Ethocel 100FP + Carbopol 934P NF (10:5)	32.35 ± 0.24	13.96 ± 0.03	1.15 ± 0.06
Ethocel 100P (10:5) + Carbopol 934P NF with HPMC	31.12 ± 0.06	12.73 ± 0.72	1.14 ± 0.75
Ethocel 100FP (10:5) + Carbopol 934P NF with HPMC	34.18 ± 0.09	14.78 ± 0.41	1.16 ± 0.67
Ethocel 100P (10:5) + Carbopol 934P NF with CMC	30.33 ± 0.15	11.71 ± 0.63	1.13 ± 0.19
Ethocel 100FP (10:5) + Carbopol 934P NF with CMC	28.26 ± 0.23	9.58 ± 0.70	1.09 ± 0.33
Ethocel 100P (10:5) + Carbopol 934P NF with Starch	33.23 ± 0.45	14.83 ± 0.09	1.17 ± 0.39
Ethocel 100FP + Carbopol 934P NF (10:5) with Starch	30.82 ± 0.68	11.45 ± 0.06	1.14 ± 0.03

**Table 3:** Physical characteristics.

Formulations	Thickness (mm, *n* = 10, Acceptable Limit 2–4 nm)	Diameter (mm, *n* = 10, Acceptable Limit 4–13 nm)	Friability (%, *n* = 20, Acceptable limit < 0.8%)	Hardness (kg/cm^2^, *n* = 10, Acceptable Limit 5–10 kg/cm^2^)	Weight Variation (mg, *n* = 20, 130–324 Acceptable Variation ± 7.5%)
Ethocel 100P + Carbopol 934P NF (10:3)	2.5 ± 0.15	8.0 ± 0.36	0.05 ± 0.40	8.2 ± 0.61	203 ± 0.38
Ethocel 100FP + Carbopol 934P NF (10:3)	2.4 ± 0.08	8.0 ± 0.08	0.08 ± 0.15	9.2 ± 0.19	202 ± 0.12
Ethocel 100P + Carbopol 934P NF (10:4)	2.5 ± 0.33	8.0 ± 0.47	0.13 ± 0.09	8.4 ± 0.25	200 ± 0.32
Ethocel 100FP + Carbopol 934P NF (10:4)	2.4 ± 0.27	8.0 ± 0.23	0.23 ± 0.35	9.6 ± 0.03	200 ± 0.21
Ethocel 100P + Carbopol 934P NF (10:5)	2.5 ± 0.49	8.0 ± 0.46	0.34 ± 0.16	8.5 ± 0.35	202 ± 0.33
Ethocel 100FP + Carbopol 934P NF (10:5)	2.4 ± 0.65	8.0 ± 0.23	0.13 ± 0.32	9.8 ± 0.06	199 ± 0.44
Ethocel 100P + Carbopol P934 NF (10:5) with HPMC	2.5 ± 0.19	8.0 ± 0.44	0.02 ± 0.28	7.7 ± 0.21	202 ± 0.16
Ethocel 100FP + Carbopol P934 NF (10:5) with HPMC	2.4 ± 0.05	8.0 ± 0.99	0.22 ± 0.31	9.4 ± 0.03	201 ± 0.25
Ethocel 100P + Carbopol P934 NF (10:5) with CMC	2.5 ± 0.07	8.0 ± 0.68	0.08 ± 0.49	9.4 ± 0.27	200 ± 0.28
Ethocel 100FP + Carbopol P934 NF (10:5) with CMC	2.4 ± 0.03	8.0 ± 0.39	0.19 ± 0.05	9.9 ± 0.17	199 ± 0.34
Ethocel 100 P + Carbopol P934 NF (10:5) with Starch	2.5 ± 0.14	8.0 ± 0.42	0.15 ± 0.43	8.5 ± 0.16	201 ± 0.53
Ethocel 100FP + Carbopol P934 NF (10:5) with Starch	2.4 ± 0.12	8.0 ± 0.28	0.07 ± 0.26	8.6 ± 0.13	200 ± 0.81

**Table 4:** Content uniformity of various formulations.

Formulations	Content Uniformity (%, *n* = 10)
Ethocel 100P + Carbopol 934P NF (10:3)	98.94
Ethocel 100FP + Carbopol 934P NF (10:3)	99.32
Ethocel 100P + Carbopol 934P NF (10:4)	98.79
Ethocel 100FP + Carbopol 934P NF (10:4)	98.56
Ethocel 100P + Carbopol 934P NF (10:5)	99.09
Ethocel 100FP + Carbopol 934P NF (10:5)	98.52
Ethocel 100P + Carbopol 934P NF (10:5) with HPMC	98.90
Ethocel 100FP + Carbopol 934P NF (10:5) with HPMC	99.00
Ethocel 100P + Carbopol 934P NF (10:5) with CMC	98.70
Ethocel 100FP + Carbopol 934P NF (10:5) with CMC	98.61
Ethocel 100P + Carbopol 934P NF (10:5) with Starch	99.43
Ethocel 100FP + Carbopol 934P NF (10:5) with Starch	99.08

**Table 5:** Drug release kinetics.

Ist-Order Kinetic		Zero-Order Kinetic		Hixon Crowell’s Erosion Model		Highuchi Diffusion Model		Power Law		
k_1_ ± SD	r^2^	k_2_ ± SD	r^2^	k_3_ ± SD	r^2^	k_4_ ± SD	r^2^	k_5_ ± SD	r^2^	N
**Losartan Potassium + Ethocel 100P + Carbopol 934P NF (10:3) Controlled Release Matrices**										
−0.354 ± 0.43	0.884	7.387 ± 0.37	0.986	0.356 ± 0.43	0.942	7.654 ± 0.54	0.987	0.012 ± 0.03	0.973	0.682
**Losartan Potassium + Ethocel 100FP + Carbopol 934P NF (10:3) Controlled Release Matrices**										
−0.395 ± 0.28	0.871	8.525 ± 0.31	0.990	0. 457 ± 0.28	0.897	7.786 ± 0.58	0.992	0.013 ± 0.44	0.945	0.633
**Losartan Potassium + Ethocel 100P + Carbopol 934P NF (10:4) Controlled Release Matrices**										
−0.351 ± 0.37	0.873	7.783 ± 0.23	0.994	0.293 ± 0.26	0.883	6.758 ± 0.69	0.991	0.016 ± 0.08	0.939	0.626
**Losartan Potassium + Ethocel 100FP + Carbopol 934P NF (10:4) Controlled Release Matrices**										
−0.393 ± 0.38	0.789	7.988 ± 0.66	0.992	0.289 ± 0.26	0.845	6.657 ± 0.55	0.994	0.018 ± 0.26	0.942	0.734
**Losartan Potassium + Ethocel 100P + Carbopol 934P NF (10:5) Controlled Release Matrices**										
−0.379 ± 0.30	0.863	8.355 ± 0.34	0.986	0.276 ± 0.53	0.990	7.769 ± 0.365	0.983	0.028 ± 0.18	0.988	0.957
**Losartan Potassium + Ethocel 100FP + Carbopol 934P NF (10:5) Controlled Release Matrices**										
−0.186 ± 0.31	0.681	8.768 ± 0.54	0.988	0.264 ± 0.59	0.978	7.786 ± 0.53	0.986	0.073 ± 0.29	0.986	0.895
**Losartan Potassium and Ethocel 100 Premium + Carbopol 934P NF (10:5) Controlled Release Matrices with HPMC**										
−0.131 ± 0.16	0.776	2.347 ± 0.11	0.782	0.116 ± 0.13	0.728	2.276 ± 0.75	0.789	0.039 ± 0.01	0.875	0.758
**Losartan Potassium and Ethocel 100FP Premium + Carbopol P934 NF (10:5) Controlled Release Matrices with HPMC**										
−0.173 ± 0.12	0.889	3.269 ± 0.66	0.898	0.198 ± 0.17	0.862	2.382 ± 0.63	0.894	0.054 ± 0.04	0.849	0.766
**Losartan Potassium and Ethocel 100 Premium + Carbopol 934P NF (10:5) Controlled Release Matrices with CMC**										
−0.145 ± 0.16	0.888	4.354 ± 0.34	0.968	0.235 ± 0.25	0.881	3.661 ± 0.28	0.956	0.016 ± 0.02	0.987	0.694
**Losartan Potassium and Ethocel 100FP Premium + Carbopol 934P NF (10:5) Controlled Release Matrices with CMC**										
−02873 ± 0.16	0.769	3.775 ± 0.36	0.982	0.143 ± 0.11	0.987	3.268 ± 0.76	0.984	0.041 ± 0.01	0.989	0.779
**Losartan Potassium and Ethocel 100 Premium + Carbopol 934P NF (10:5) Controlled Release Matrices with Starch**										
−0.298 ± 0.13	0.879	2.354 ± 0.55	0.980	0.176 ± 0.12	0.985	3.721 ± 0.27	0.979	0.045 ± 0.01	0.976	0.737
**Losartan Potassium and Ethocel 100FP Premium + Carbopol 934P NF (10:5) Controlled Release Matrices with Starch**										
−0.292 ± 0.78	0.889	4.359 ± 0.22	0.985	0.154 ± 0.11	0.978	3.481 ± 0.25	0.972	0.049 ± 0.18	0.984	0.854

**Table 6:** Results of *f*_1_ and *f*_2_.

Test Formulation versus Reference Cardaktin^®^ Tablet	*f*_1_ Values (Acceptable Limit 1–15)	*f*_2_ Values (Acceptable Limit 50–50)
Ethocel 100P + Carbopol 934P NF (10:3) CR Matrices versus Cardaktin^®^ Tablet	42.73	17.99
Ethocel 100FP + Carbopol 934P NF (10:3) CR Matrices versus Cardaktin^®^ Tablet	52.64	13.47
Ethocel 100P + Carbopol 934P NF (10:4) CR Matrices versus Cardaktin^®^ Tablet	53. 22	12.38
Ethocel 100FP + Carbopol 934P NF (10:4) CR Matrices versus Cardaktin^®^ Tablet	56. 48	10.16
Ethocel 100P + Carbopol 934P NF (10:5) CR Matrices versus Cardaktin^®^ Tablet	38.16	20.76
Ethocel 100FP + Carbopol 934P NF (10:5) CR Matrices versus Cardaktin^®^ Tablet	41.69	17.78
Ethocel 100P + Carbopol 934P NF (10:5) with HPMC CR Matrices versus Cardaktin^®^ Tablet	46.53	14.06
Ethocel 100FP + Carbopol 934P NF (10:5) with HPMC CR Matrices versus Cardaktin^®^ Tablet	48.54	13.22
Ethocel 100P + Carbopol 934P NF (10:5) with CMC CR Matrices versus Cardaktin^®^ Tablet	56.48	11.36
Ethocel 100FP + Carbopol 934P NF (10:5) with CMC CR Matrices versus Cardaktin^®^ Tablet	47.78	14.98
Ethocel 100P + Carbopol 934P NF (10:5) with Starch CR Matrices versus Cardaktin^®^ Tablet	45.34	14.04
Ethocel 100FP + Carbopol 934P NF (10:5) with Starch CR Matrices versus Cardaktin^®^ Tablet	48.76	13.23

## Data Availability

Requests to access the datasets should be directed to the corresponding author.

## Sample Availability

Samples of the compounds are available from the authors.
